# BioUSeR: a semantic-based tool for retrieving Life Science web resources driven by text-rich user requirements

**DOI:** 10.1186/2041-1480-4-12

**Published:** 2013-05-01

**Authors:** María Pérez, Rafael Berlanga, Ismael Sanz, María José Aramburu

**Affiliations:** 1 Department of Computer Science and Engineering, Universitat Jaume I, Castellón, Spain; 2 Department of Computer Languages and Systems, Universitat Jaume I, Castellón, Spain

**Keywords:** Resources discovery, Semantic annotation, Information retrieval, Life science

## Abstract

**Background:**

Open metadata registries are a fundamental tool for researchers in the Life Sciences trying to locate resources. While most current registries assume that resources are annotated with well-structured metadata, evidence shows that most of the resource annotations simply consists of informal free text. This reality must be taken into account in order to develop effective techniques for resource discovery in Life Sciences.

**Results:**

BioUSeR is a semantic-based tool aimed at retrieving Life Sciences resources described in free text. The retrieval process is driven by the user requirements, which consist of a target *task* and a set of *facets* of interest, both expressed in free text. BioUSeR is able to effectively exploit the available textual descriptions to find relevant resources by using semantic-aware techniques.

**Conclusions:**

BioUSeR overcomes the limitations of the current registries thanks to: (i) rich specification of user information needs, (ii) use of semantics to manage textual descriptions, (iii) retrieval and ranking of resources based on user requirements.

## Background

In this section we introduce the context of our work and our motivation. Then, we summarize the related work and we present the rationale of our proposal.

### Introduction and motivation

In recent years, the research activity of the Life Sciences community has produced a huge amount of data as well as many resources and tools to manage it. Nowadays, the success of many research tasks in the Life Science depends on the integration of the proper resources and tools which can be accessed through the Internet. As an example task, let us consider the combination of DNA sequencing with reference databases available on the web [1], which is followed by complex analysis workflows that rely on highly specific algorithms, often available as web services [2]. In this scenario the amount of data produced and consumed is prodigious, and the sheer amount of available resources to manage research data is a source of severe difficulties. In this work, we consider web resources as any application, information source, service or site that can be identified or handled in the Web and which provides functional and processable metadata about its functionality and features.

A web resource registry is a repository in which providers register their resources (e.g., web services, datasets and so on) with the aim that other users can discover and use them. As a result of different research efforts, currently there are many registries with resources related to Life Sciences. Table 1 shows the comparison of some of the most frequently used ones. This comparison is based on how users specify their requirements, the type of search, the use of semantics in the discovery process and functionalities related to resource composition.

**Table 1:** Comparative table of registries of web resources in Life Sciences

**Registry**	**User**	**Discovery**	**Semantics**	**Composition**
	**requirements**			
Feta [3]	Keywords	Input, output,	Manually	N/A
	from ontology	operation type,		
		task		
BioMoby [4]	Keywords	Resource type, I/O	Resource type,	N/A
			object type	
EMBRACE [5]	Keywords	String matching	Syntactically annotated	N/A
			with BioXSD	
BioCatalogue [6]	Keywords	String matching,	Categories, some tags	N/A
		categories, filters		
SSWAP [7]	Keywords,	RDF	Third-party ontologies,	N/A
	Resource		reasoning	
	Query Graph			
Magallanes [8]	Keywords	String matching in	N/A	Yes
		data type,		
		resource type		
myExperiment	Keywords	String matching,	Tags	N/A
[9]		filters		
Taverna [10]	Keywords	String matching,	BioMoby metadata	Workflow
		ontology concepts		composition
SADI [11]	SPARQL	RDF	Third-party ontologies	Yes

This comparative table presents the main characteristics of the most popular registries in Life Sciences.

Most of them provide search based on keywords or filters, which implies that users have to know the vocabulary used to describe the web resources and, moreover, the success of the search depends on the available information about the resource.

Another limitation is that most of the registries assume the availability of well-defined metadata about the features of the resources, e.g., input and output data types. However, in open registries relevant information about the features of a resource (e.g., input/output data types, method and species involved) is usually described in the textual description and, therefore, it is not expressed as relevant metadata.

In this paper we present BioUSeR (Bioinformatics User-driven discovery of Semantically-enriched Resources), a tool to assist the researcher in the discovery of the most suitable resources to her information needs and that overcomes the limitations presented above by: *(i)* allowing the user to provide a text-rich specification of her requirements including the target task and important features of the resources, *(ii)* exploiting text-rich descriptions to discover and classify relevant information about the resources to better characterize them and use this information as facets for the search, *(iii)* using semantic annotation to allow mappings between information written in free text.

### Related work

Next, we provide a brief description of the features of current web resource registries in the Life Sciences.

Feta [3] is a faceted retrieval system for Life Sciences resources in which the user queries are based on the input and output data types, the method or type of an operation, or a phrase contained in the description of an operation. Feta requires that resources have to be manually annotated with the ^*my*^Grid ontology, and then, searches must be also based on this ontology. In this work, ranking is not applied because they argue the registry is very small.

BioMoby [4] is an open-source research project whose aim is to implement a web-resources registry to facilitate the discovery and sharing of biological data. Resources are registered in MOBY Central by using a model that allows search and retrieval based on object and resource hierarchies. Users may request a search for available resources based on their input, output, resource type or authority by using keywords.

EMBRACE Resource Registry [5] is a Life Sciences web resources registry with built-in resource testing. Resources are syntactically annotated using BioXSD [12]. The search is based on the string matching of keywords. This registry is the prelude to BioCatalogue.

BioCatalogue [6] is a Life Sciences registry that provides a common interface for registering, browsing and annotating Life Sciences web resources. Web resources in BioCatalogue can be annotated with categories, tags and descriptions. These annotations are manually provided by the resource providers and the user community plus some monitoring and usage analysis data obtained automatically by BioCatalogue servers. However, at the moment, most of these annotations are expressed as free text without following any controlled vocabulary. The resource discovery is mainly based on both keyword search and filtering mechanisms. Filters can be applied over: resource type, provider, submitter and country. To enhance its accessibility and usability, BioCatalogue is indexed by search engines such as Google ^*TM*^. It also provides a programmable API which is used by third-party applications such as Taverna [10].

SSWAP [7] proposes an architecture, a protocol and a platform to semantically discover and integrate heterogeneous disparate resources on the web. Unfortunately, this approach heavily relies on the provided metadata, which is usually poorly described. SSWAP provides two types of searches: keyword search and resource query graph. The keyword search presents the problems related with the selection of keywords and the lack of useful metadata. Resource graph query requires training to learn how to build the graph and how to express the queries with this format, which means a high effort for those users not familiar with these technologies.

Magallanes [8] is a library of algorithms aimed at discovering bioinformatics web resources. The search is based on a Google ^*TM*^-like approach, in which the user keywords are matched to metadata descriptions improved by the *Did you mean...?* algorithm which helps the user to build the query. Search can be performed on data type, resource and resource type fields and it is improved by a learning process from users feedback. Moreover, Magallanes provides a way of composing compatible resources into workflows.

myExperiment [9] is a Life Science repository whose main resources are workflows but other research objects can also be registered in it. It has been developed in the same project as BioCatalogue. The workflows can be annotated with tags, a description, object type and other information about the provider like the country, etc. The search is based on keywords and filters over the previous fields. myExperiment provides also information about the popularity of the resource, such as the times the resource has been viewed or downloaded, and a rating scale that reports about the quality of the resource.

Taverna [10] is a workflow construction environment and execution engine designed to support *in silico* experiments developed by the European Bioinformatics Institute (EBI) and University of Manchester. Taverna is part of ^*my*^Grid project and so is aligned to BioCatalogue and myExperiment. It is able to build complex workflows, to execute them and to display the different types of results. The user selects the resources with a keyword search. Taverna contains the BioMoby resources and, therefore, the input and output data types are well defined. Other resources can be imported to Taverna.

SADI [11] framework is a registry that uses standard-compliant Semantic Web Resource design patterns that simplify the publication of resources and their subsequent discovery in domains such as bioinformatics. Providers have to follow SADI conventions to publish their resources, and users have to create SPARQL queries in order to discover the desired resources, which implies that users have to know the SPARQL query language, which supposes an extra effort for example for biologists, that may not be experts in these technologies.

### Rationale

From the related work presented above, we can conclude that these registries limit the users in the specification of their requirements since they have to use specific keywords usually expressed in an application ontology like ^*my*^Grid or create queries in specific query languages such as SPARQL. Moreover, most of them base the discovery on specific metadata such as input and output data types assuming them available but that rarely appear. As the above text shows, open-metadata registries (e.g., BioCatalogue and SSWAP) hardly provide rich metadata as stand-alone applications do (e.g., Magallanes). It is also worth noting that none of them exploits the description of the resources in order to find relevant metadata of the resources, with the consequent loss of relevant information.

To solve these limitations, there are some key aspects that must be addressed: the non-intuitive specification of the users’ requirements in current registries, the under-use of available standards in the description of web resources, and the lack of automatic mechanisms to determine the degree of suitability of the discovered resources. As it is shown in this paper, BioUSeR addresses these aspects by using both semantic annotations as a normalization process consisting in the association of formal knowledge to the available text descriptions, and information extraction techniques in order to obtain information about the facets from the annotated task descriptions.

## Results and discussion

In this section we present BioUSeR, a prototype that has been developed to show the usefulness of our approach, and we demonstrate its usefulness with a case study. Then, we evaluate it and, finally, we discuss the results.

### BioUSeR

We have developed a prototype called BioUSeR (Bioinformatics User-driven discovery of Semantically-enriched Resources) that assists users of web resource registries in each step of the retrieval process, from the requirements specification until the resource selection. This prototype is user-oriented and one of its main characteristics is that it allows the user to configure all the process in an easy and intuitive way.

The current prototype is focused on the Bioinformatics domain and we have selected BioCatalogue as the reference web resource catalogue. However, other catalogues (e.g., SSWAP) can be easily integrated by only registering the information of the resources in our repository. BioCatalogue contains 2081 registered resources (as of November 2011). Although some resources are described through a set of predefined categories, most of them have no metadata and just provide a free text description and/or the web resource documentation. Some resources do not provide any kind of information but just the URL to their web sites. For these cases, we have downloaded the web site main pages and used them as the resource descriptions. We remark that these limitations motivate the use of our approach.

**Figure 1:**
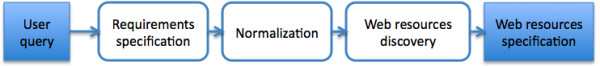
**Overview of the proposed approach.** The approach is divided on three phases: requirements specification, normalization and web resource discovery.

The retrieval process in BioUSeR is divided in three phases, as shown in Figure 1: (i) requirements specification, (ii) normalization and facets extraction and (iii) resource retrieval and selection. Next, we present a case study to show the results of each one of these phases.

### Case study

To demonstrate the usefulness of BioUSeR, we use it to develop a Life Science case study extracted from [13]. The case study concerns biological research that analyzes the presence of specific genes involved in the genesis of Parkinson’s disease, called LRRK2 genes, in different organisms. Next, each step of the process is explained with a brief description and a snapshot of BioUSeR. 

**Figure 2:**
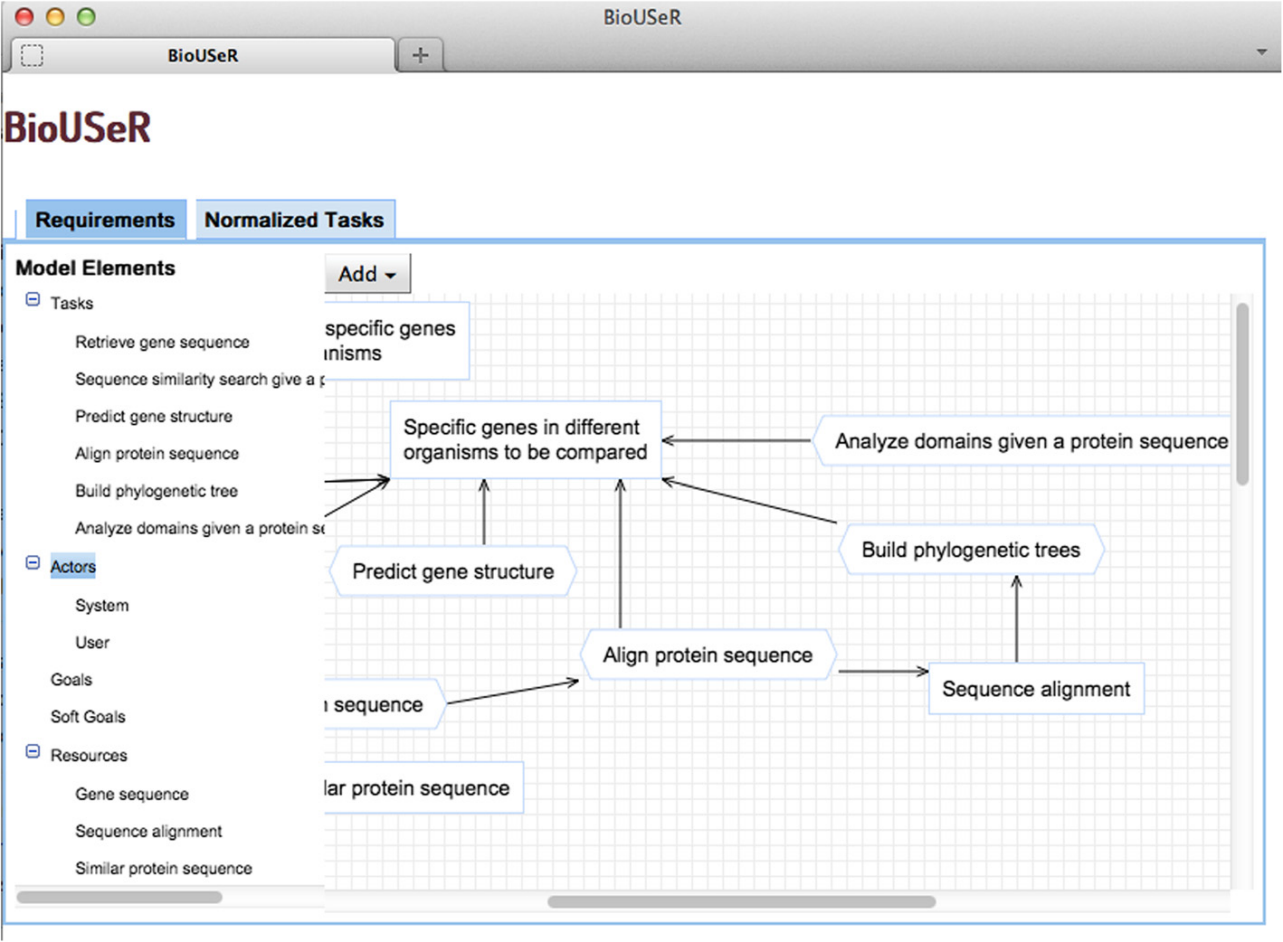
**Requirements model.** This figure shows the requirements model defined by a user who wants to compare specific genes in different organisms.

1. **Requirements specification**. The user needs to compare specific genes in different organisms as part of a study of the presence of the *LRRK*2 genes in the organism *N*.*Vectensis*, since previous studies have shown that this is a key organism to trace the origin of these genes. With BioUSeR, the user’s query would be a requirements specification consisting of the goal *specific genes in different organisms to be compared* plus a set of tasks specified in a requirements model by using natural language descriptions. In this case, the user defines five tasks in order to achieve the main goal: (*i*) to search similar sequences given a protein sequence, (*ii*) to predict the gene structure; (*iii*) to align protein sequences; (*iv*) to build a phylogenetic tree; and (*v*) to analyze the domains in protein sequences. Figure 2 shows a fragment of the requirements model specified by the user in which the tasks “predict gene structure”, “align protein sequences”,“build phylogenetic trees” and “analyze domains given a protein sequence” are shown.

**Figure 3:**
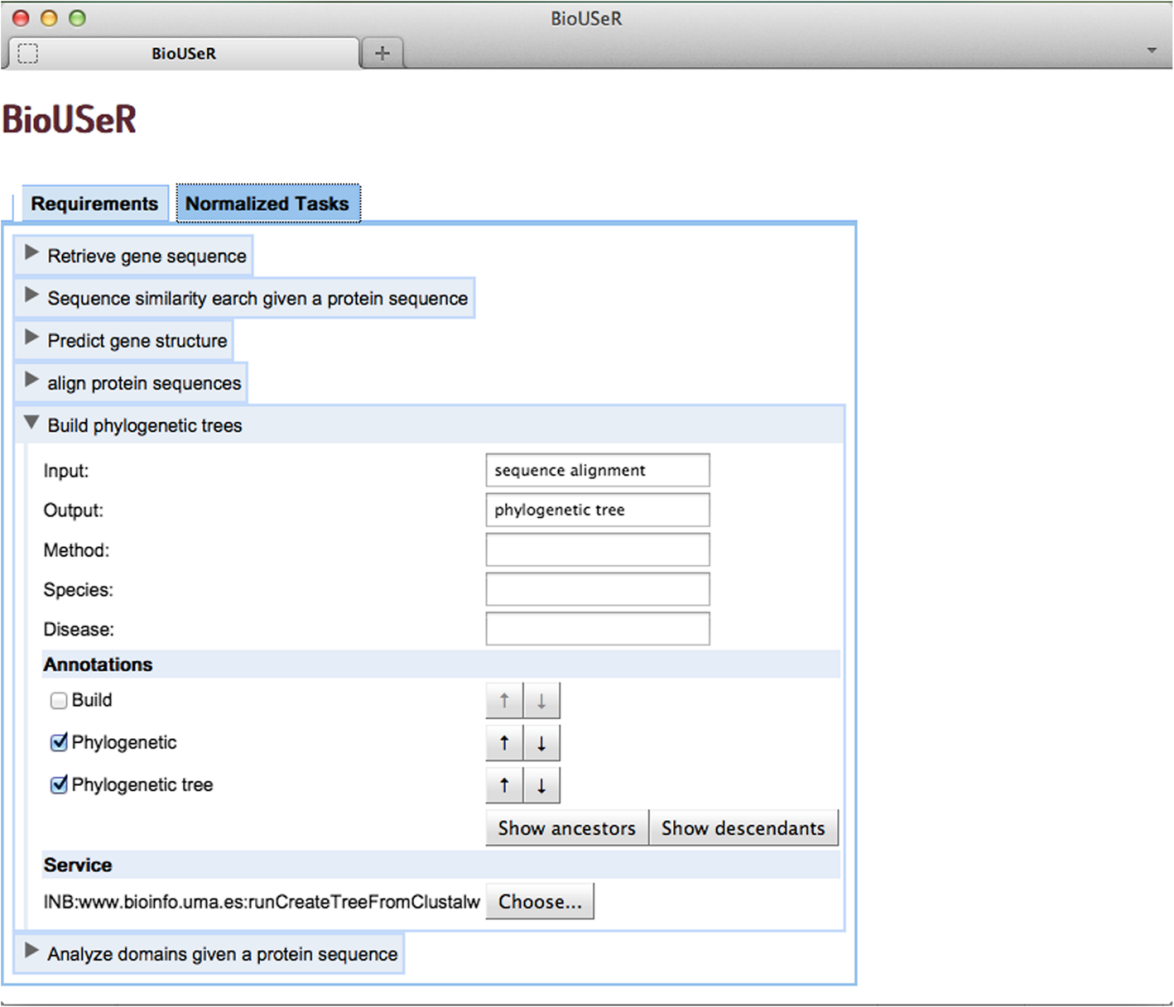
**Information of a normalized task.** This figure shows the information of the user-defined tasks once they have been normalized. For each task, it shows the facets values, the semantic annotations and the selected resource.

This case study also shows that in the requirements specification the value of the facets can be: *(i)* implicitly described in the task, e.g. “search similar sequences given a protein sequence”, *(ii)* determined directly as the value of the facet as happens in the output of the task “build phylogenetic tree” as it is shown in Figure 3, or *(iii)* implicitly determined by the dependencies between tasks, e.g., the task “build phylogenetic trees” has as input the data generated by the task “align protein sequences”.

2. **Normalization and facets extraction**. Once the user has completed her requirements specification, the next step is to normalize and analyze it. First, the descriptions of the tasks are semantically annotated with the unified knowledge resources (KR) and BioUSeR provides the user with the concepts of the annotations. Then, the user can reject those that are not appropriate due to ambiguity or to errors of the annotator. Moreover, BioUSeR also allows the user to select the ancestors or descendants of these concepts. Finally, the requirements specification is translated into a semantic vector that contains the selected concepts. In Figure 3, the *Annotations* section shows the annotations of the task “build phylogenetic trees” and the selection dialogue prompted to the user.

Additionally, from the annotated task descriptions, information about the facets is extracted by using the information extraction patterns shown in Table 3. In the task “search a similar sequence given a protein sequence”, the input type *protein sequence* is implicitly described and it is extracted by the extraction pattern *given noun-phrase*.

3. **Resources retrieval and selection**. The retrieval of the most appropriate resources is carried out by a semantic mapping between the semantic vectors of the requirements specification and the semantic vectors of the resources.

At the end, the user gets a ranked list of resources for each task and can visualize metadata about each resource which helps her in the selection of the most appropriate ones. The *Resource* section of Figure 3, the first ranked resource is selected by default, but the user can select any other resource by pressing the *Choose* button. Table 2 shows the information for each task, the defined facets and the resources selected. For the task “build phylogenetic tree”, the selected resource is the fifth ranked resource, but it is the one that best fulfils the task and the facets required by the user.

**Table 2:** Example of extraction patterns for facets

**Facet**	**NP**	**R**
Input	Inputs?	(is |are)
	-	Given
		Taking
	(((of)? E)+ | it)	gets
Output	Outputs?	(is |are)
	(((of)? E)+ |it)	Constructs
		Finds
		Retrieves
		Calculates
		Contains
		Produces
		Extracts
		Returns
Method	(((of)? E)+ | it)	Maps
		Executes
		Performs
		Implements
		Applies
		Applying
		Runs
		Running
		Based on
		Computes
		Carries out
		Processes

It shows some of the extraction patterns specified for the facets: input, output and method.

**Table 3:** Results for the case study

**Task**	**Facets**	**Resource**	**Rank**
Retrieve gene sequence	Input: gene	getColiCardIDs_by_	1
	Output: sequence	InteractingPartnersResource	
Search similar sequences	Input: sequence	Database of Protein	1
	Output: blast report	Subcellular Localization Resource	
Predict gene structure	Input: gene	GlimmerResource	1
	Output: gene model		
Align protein sequences	Input: protein sequence	T-Coffee	1
	Output: sequence alignment		
Build phylogenetic trees	Input: sequence alignment	INB:http://www.bioinfo.uma.es:	5
	Output: phylogenetic tree	runCreateTreeFromClustalw	
Analyze domains	Input: protein sequence	INB:inb.bsc.es:parseRulesFromMotif	1

This table shows the selected resources for each user-defined task.

BioUSeR assists and is assisted by the user during the whole retrieval process, from the requirements specification until the resources selection, taking advantage of her knowledge and expertise on the field, and providing her with useful information like annotation concepts, resources metadata and ranked lists of resources. We want to remark that the user guides each step: selecting the appropriate concepts, choosing the most suitable resources or even redefining the initial requirements. Moreover, the user can also specify additional features of the desired resources. The use of facets improves the suitability of the results since the final list is ranked according to the task and to the facets.

### Evaluation

In this section, we first evaluate the effectiveness of the discovery system and, then, we make a more specific analysis of the facets extraction method. Finally, we further evaluate our system by comparing it to BioCatalogue, one of the most popular open registries in Life Science.

#### BioUSeR evaluation

The evaluation of BioUSeR has been carried out by executing a set of heterogeneous queries (i.e. task description examples) that captures different ways to describe bioinformatics tasks, thus reflecting the variability in the users’ information needs. The query pool [14] has been created by selecting more than 250 short descriptions extracted from other Life Sciences resource catalogues such as OBRC [15] and ExPaSy [16].

These queries have been evaluated over a gold standard (GS) due to the difficulties to determine the whole set of relevant results for each query. The GS [17] has been built with 443 resources (out of 2081 registered resources), but only for seven base tasks that can be unambiguously related to BioCatalogue categories. Moreover, we have manually revised it in order to ensure the quality of the final set.

Table 4 shows the precision, the recall and the F-measure of the results obtained by executing the queries from the query pool. These results show that the top-ranked results are, in most cases, appropriate for the user’s requirement and, moreover, the recall shows that most of the relevant resources are provided to the user.

**Table 4:** BioUSeR evaluation

**Base task**	**P@5**	**P@10**	**P@20**	**P**	**R**	**F**
Search proteins with a functional domain	0.79	0.76	0.73	0.45	0.63	0.53
Search similar sequences	0.91	0.85	0.77	0.22	0.72	0.33
Analyze transgenic model organism	0.93	0.94	0.89	0.58	0.89	0.7
Find genes with functional relationships	0.78	0.75	0.66	0.34	0.39	0.36
Predict structure	0.91	0.91	0.85	0.47	0.29	0.36
Analyze phylogeny	0.8	0.8	0.79	0.52	0.36	0.43
Align sequences	0.73	0.76	0.74	0.62	0.3	0.41

This table shows the precision, recall and F-measure of the results obtained for the queries of the query pool, associated to the 7 base tasks of the gold standard (GS). It also includes the precision for the top-5, top-10 and top-20 results.

Moreover, we have also evaluated the use of semantics in the normalization process in order to know how the semantic annotations improve the search. To that end, we have evaluated the results of the queries from the query pool without semantic annotations, that is, the retrieval is based on words and not in concepts. The precision is in average 32% and the recall is in average 38%. Therefore, we can conclude that semantic annotations improve significantly the web resources discovery.

#### Facets extraction evaluation

In order to evaluate the quality of extracted facets, we have set up a GS data set with information about the facets of the resources registered in BioCatalogue. BioCatalogue allows users to assign tags to resources in order to describe some aspects of them. Currently there are 855 tags for describing 2081 web resources. This GS has been built as follows. For input/output facets, we have automatically selected the tags assigned to the input/output descriptions. For the other facets, we have manually classified the tags into method, species and disease facets. A summary of the number of tags and involved resources for each facet is shown in Table 5. Notice the low number of resources having tags for the input/output descriptions in BioCatalogue, which confirms the lack of processable metadata in this kind of open registries.

**Table 5:** Facets gold standard

**Facet**	**Tags**	**Resources**
Input	52	48
Output	47	48
Method	135	434
Disease	7	5
Species	27	61

This table shows for each facet: the number of BioCatalogue tags in the facets gold standard (GS) and the number of resources that are tagged with them.

Table 6 presents the number of concepts that have been automatically extracted for each facet by using extraction patterns and semantic types, and the number of resources that are annotated with these concepts.

**Table 6:** Facets extraction evaluation

**Facet**	**Concepts**	**Resources**	**Precision**	**Recall**	**F-measure**
Input	259	399	0.69	0.93	0.73
Output	266	274	0.6	0.94	0.64
Method	136	210	0.44	0.53	0.35
Disease	142	144	-	-	-
Species	292	287	-	-	-

This table shows the number of concepts that our approach has automatically extracted for each facet and the number of resources that are annotated with those concepts. Moreover, for the input/output and method facets the precision, recall and F measures are shown. These measures have been calculated for the automatically extracted information with respect to the GS. The disease and species facets have not been evaluated because of their poor representation in the GS.

The facet method is the most utilized by users for describing resources, whereas the disease facet is seldom described via tags. However, our tool detects a greater number of values for these facets, although it works worst for the method facet. The latter issue is due to the poor coverage of the reference ontologies with respect to bioinformatics algorithms and methods.

We have also evaluated the precision, recall and F-measure of the input, output and method extracted facets with respect to the GS and the results are shown in Table 6. We have not evaluated these measures for the disease and species facets due to their poor representation in the GS.

#### Comparison with BioCatalogue

With the aim of validating our approach, we compare it with the BioCatalogue search engine. We have selected BioCatalogue for several reasons: first, nowadays it is one of the most popular open registries in Life Science; second, BioUSeR has been evaluated with a GS set up with the resources registered in BioCatalogue and, third, because BioCatalogue provides an API that allows users to query it programmatically. BioCatalogue provides two types of search: (i) keyword-based search and (ii) navigational-based search using categories. Each type of search has been evaluated separately. In both cases, the results have been evaluated using the GS described above. Next, we describe with more details each evaluation.

Keyword search is based on string matching techniques that use all the information available about the resources. This type of search supposes an extra effort to the user since she has to summarize her informational needs in a set of words and these words have to make a complete matching with the words in the resource information. For instance, the query *metabolic pathways* does not retrieve any resource, however its singular form *metabolic pathway* retrieves some resources. Table 7 shows the precision, recall and F-measure of the results obtained by manually built keyword queries that try to express the informational needs described in the requirements. This table also shows the cost of edition, that is, the average number of failed queries we have executed before getting some results, which is in average 2.89, and the number of keywords per query, which is in average 2.94. Considering the precision and the recall, keyword queries do not provide good results considering user’s requirements. Our approach presents better precision and recall, that is, it retrieves more relevant results, moreover, without transforming the original requirements.

**Table 7:** BioCatalogue keyword search evaluation

**Task**	**P@5**	**P@10**	**P@20**	**P**	**R**	**F**	**Edition**	**Keywords**
							**cost**	
Search proteins with	0.4	0.41	0.41	0.41	0.02	0.04	2.45	3.4
a functional domain								
Search similar sequences	0.4	0.4	0.4	0.36	0.07	0.12	2.87	3.8
Analyze transgenic	0.74	0.71	0.71	0.71	0.17	0.27	3.25	2.94
model organism								
Find genes with	0.27	0.26	0.27	0.26	0.04	0.07	3.15	2.13
functional relationships								
Predict structure	0.67	0.66	0.65	0.64	0.04	0.07	3.27	2.93
Analyze phylogeny	0.18	0.2	0.2	0.18	0.01	0.02	2.8	2.56
Align sequences	0.72	0.72	0.75	0.69	0.07	0.13	2.48	4.16

This table shows the evaluation of the keyword-based search of BioCatalogue. The evaluation has been made using the queries in our GS and precision, recall and F measures have been calculated for each topic. The edition cost is the cost of translating the requirements into keywords queries, which corresponds to the number of failed queries executed before getting results. Finally, keywords is the average number of keywords of the successful queries.

Navigational search allows the user to navigate through the BioCatalogue taxonomy of categories, i.e., the most common bioinformatics tasks. When the user selects a category, BioCatalogue filters the resources that are tagged with that category. BioCatalogue allows to select several categories, but it does not combine navigational search with keyword search. An important limitation of this search is that it does not retrieve those resources that are not categorized, even when the category appears in their description expressed in natural language. Another limitation is the broadness of the categories, which does not allow the user to express more specific tasks. To evaluate the navigational search, we have manually selected the most suitable categories for each query in the query pool. Table 8 shows the precision, recall and the F-measure of the results, and the cost of edition of the queries. In this type of search, the cost of edition is represented by the depth of the category in the taxonomy and the number of siblings of the selected category, describing in this way the steps required to select the most appropriate category. The higher the depth, the more specific the category is. On average, the precision is high but it is not possible to know if the retrieved results perform the specific task described in the requirement. Our approach presents a lower precision but a higher recall, that is, it retrieves relevant resources that the navigational search does not retrieve, e.g., those that are not categorized. Moreover, our approach retrieves resources that perform the specific tasks described in the requirements, which is not possible with the navigational search.

**Table 8:** BioCatalogue navigational search evaluation

**Task**	**P@5**	**P@10**	**P@20**	**P**	**R**	**F**	**Edition cost**
Search proteins with a functional domain	0.92	0.92	0.82	0.75	0.15	0.25	2.67/3.3
Search similar sequences	1	1	1	1	0.3	0.46	2.0/4.25
Analyze transgenic model organism	0.8	0.9	0.95	0.94	0.4	0.56	0.03/10.77
Find genes with functional relationships	0.91	0.95	0.89	0.89	0.26	0.4	1.0/3.0
Predict structure	0.87	0.93	0.96	0.9	0.1	0.18	2.29/3.42
Analyze phylogeny	0.8	0.88	0.88	0.88	0.03	0.06	0.0/11.0
Align sequences	0.98	0.99	0.99	0.99	0.06	0.11	2.94/2.1

This table shows the evaluation of the BioCatalogue navigational search based on categories. The evaluation has been made using the queries in our GS and the average precision, recall and F-measure have been calculated for each topic. The cost of edition of the queries is represented by the depth of the category in the taxonomy of categories and the number of siblings of the selected category.

Another important limitation of both types of search is that they do not provide a ranked list, so the user has to manually check all the results. Nevertheless, BioUSeR provides the user with a ranked list of resources depending on their suitability to the requirement.

Regarding facets, BioCatalogue allows the user to search by introducing input or output data examples, retrieving those resources that require or produce these data. However, they do not combine this search with the others and, therefore, the user cannot specify which task she wants to perform. In BioUSeR, the user can describe the required functionality and information about the facets in the same query.

We can conclude that our approach improves BioCatalogue search engine by using natural language queries, which describe the task and the features of the resources, avoiding the selection of keywords or general categories that do not describe specific tasks. Moreover, the semantic annotation addresses the problem of using different vocabularies or string mismatchings.

### Discussion

Most of the current registries base the discovery of resources on keywords or concepts coming from their own ontologies that describe the tasks or the input and output data types. There are approaches that use string matching techniques with all the available information, and others that are based only on the metadata of the resources. The former assume that users know the vocabulary with which the resources are described, since they have to specify the correct keywords. The latter do not take into account all the information available in the resource descriptions and documentation which are expressed in natural language, so they assume that resources are provided with useful metadata and, as we have mentioned before, this does not happen in current open-metadata registries for Life Sciences.

Our approach allows the user to specify her information needs as rich textual descriptions. While current registries do not allow the user to combine in the same query information about the task and the features of the resources, in BioUSeR the user can provide a description of the functional tasks she needs to be executed to achieve a goal together with the set of relevant features that the retrieved resources must have. Currently, BioUSeR supports the following facets: input and output data types, the method and the disease and species involved, but a new facet can be easily added by only determining its adequate information extraction patterns and the involved semantic types. Considering the features of the resources, we bring to our tool the well-known advantages of using facets to restrict the search and enhance the efficiency and precision of current information retrieval systems [18].

As a result, BioUSeR makes possible that users without any special training can specify, with rich textual descriptions, all the features of the required solution for their information needs. BioUSeR allows this kind of search because of the normalization of data.

Moreover, most of the resources metadata are expressed in textual descriptions and few well-defined metadata are available on open registries. As many Life Science researchers recognize, to manually describe their resources by using standards is a very complex task that usually produces incomplete and imprecise descriptions, being this the reason for which almost always they prefer to describe them by means of free texts with non-standard vocabularies. In BioUSeR, the user requirements specification and all the available resources metadata are semantically annotated with widely accepted Life Science ontologies such as UMLS and myGrid. Additionally, information extraction techniques [19] are used to identify in the resources metadata relevant information about the set of facets supported by the system. BioUSeR looks for information about the facets in all the available metadata of the resource, and not only on specific fields as most current registries search engines do. The automatic normalization enriches the system information with formal knowledge and provides two main benefits to the system: (i) it avoids the problem of the use of specific vocabularies and (ii) it allows the system to exploit all the available information of the web resources independently of the characteristics of the metadata.

BioUSeR retrieves the resources by the semantic mapping of the normalized user requirement specification and the normalized resources metadata, and provides the user with a ranking of the retrieved resources, while current registries only provide a list. Both the retrieval and the ranking of resources are driven by the functional task and the set of user-defined facets. Thus, for each task described in the requirements specification, the system prompts to the user a short ranked list of web resources that could be used to execute it. Then, the user can easily select a resource for each task and to define a sequence of resources that can be seen as a workflow specification. In order to assist the user in the selection, the available metadata of each resource can be visualized. As the discovery process is a cyclic process, if some of the retrieved resources are not considered adequate for the user requirements, the user can modify the initial requirements specification so that alternative resources can be explored.

## Conclusions

In this paper we present BioUSeR, a tool that assists researchers in Life Sciences in the discovery of the most appropriate web resources for their well-defined requirements. With BioUSeR, users can easily find out web resources that were previously unknown to them because fell out of the scope of their main field of interest, or were poorly categorized with existing tags.

BioUSeR assists all kinds of users from the requirements specification until the selection of the most appropriate resources, not only by allowing the customization of the queries, but also by making the specification of the information needs easier to non-expert users.

The main novelty of BioUSeR with respect to existing registries is that it deals with text-rich descriptions of the registered resources apart from the provided metadata. For this purpose, BioUSeR applies automatic semantic annotation and information extraction processes. As a result, this tool automatically generates two kinds of metadata: semantic annotations and facet-value pairs. Thus, BioUSeR aims to create metadata that are useful to fulfill the user requirements, which are usually stated as free text descriptions and facet-based requests.

Future work will be mainly focused on improving both the annotation system and the extraction of facet-values. The annotation system needs to be extended with new knowledge resources containing specific Bioinformatics algorithms and methods that are now poorly covered by the selected ontologies. Also, it is necessary to treat ambiguous annotations that can produce noise in the retrieval of resources. As for the extraction of facets, an automatic method to find out relevant patterns for a facet should be designed. In this way, the definition of new facets and its inclusion into the tool will be even easier. Another interesting issue for future work is to study new methods for re-ranking retrieval results according to the existing relations between tasks. The main aim of this re-ranking is to improve the compatibility between the retrieved resources of each task. Finally, our final aim is to build an unified repository with existing ones (e.g., BioCatalogue, SSWAP, myExperiment and so on), and integrate it with Taverna [20] through BioUSeR.

## Methods

Our approach consists of three phases as depicted in Figure 1. The main purpose of our method is to normalize both the user requirements and the web resources metadata in order to compare them and to discover the web resources that best match the user’s needs. In this section we explain the methods and techniques applied at each phase.

### User requirements specification

Most current registries, as shown on Table 1, only provide keyword search or searching by filters. In this kind of registry, the users find limitations when specifying their requirements, e.g., the selection of the words to make the search, the available information of specific fields and so on. However, due to the experience and the knowledge users have on their research fields, they can easily provide natural language descriptions of their requirements and the tasks that would be manually performed to meet them. In our approach, we have adopted a hierarchical model to specify user requirements in a formal way so that they can be automatically used in the subsequent phases of the resource retrieval process.

User requirements are represented by means of goals and task elements in a formal specification called the *Requirements model*. This requirements specification is based on the *i*∗ formalism [21,22], which is both a goal-oriented and an agent-oriented language. We use this framework because it provides the functionality required to obtain a formal specification of the user’s requirements without taking into account the characteristics of the system. The goal and the task elements of the *Strategic Rationale* (SR) model of the i* framework capture the user’s information requirements and the steps to achieve them. This model generalizes the work in [23] to allow the specification of the user requirements in the context of finding appropriate similarity measures for XML data.

To better describe the desired resources, the user can specify additional features of the resources by determining values for the facets of interest of each task. A faceted search system presents users with key-value metadata that is used for query refinement. In our approach, we propose a faceted search to discover the resources that best cover the user-defined facets, more specifically: the input and output types, the method, the diseases and the species involved in the resources. It is worth noting that the set of facets can be easily extended to cover other user requirements.

Additionally, thanks to the hierarchical structure of the Requirements model, in case a task has not explicitly defined the input/output types, they can be automatically set since they can be implicitly determined by the previous/next related tasks. In this way, the model is describing the sequence of tasks that would execute the functionality required by the user.

### Normalization

User requirements can be easily described when the user can express them in natural language, without the limitation of using specific vocabularies as in most web resources discovery approaches. Unfortunately, natural language presents heterogeneity, ambiguity and implicitness issues, which make them hard to process automatically. In our approach, we use automatic semantic annotation (SA) to normalize textual descriptions of user requirements and web resources with respect to a set of reference knowledge resources.

SA can be seen as the process of linking the *entities* mentioned in a text to their *semantic descriptions*, which are usually stored in knowledge resources (KRs) such as thesauri and domain ontologies [24]. Former approaches to SA were mainly guided by users (e.g., [25]) through seed documents, manually tagged examples or ad hoc extraction patterns. However, in our scenario, we require that the SA process is fully automatic and unsupervised. This is because the volume of data to be processed is huge and the set of possible user requirements is unknown a priori. There are few approaches performing fully unsupervised SA, and they are mainly based on dictionary look-up methods or adhoc extraction patterns (see [26] for a review of SA concepts and approaches).

Our SA process consists of three main steps. In the first step, the KR is processed to generate a lexicon, which contains lexical variants with which each concept is expressed in the written texts. We denote the set of variants of a concept *C* as *lex*(*C*). The second step consists of applying some *mapping function* between the text chunks likely to contain an entity and the KR’s lexicon, in order to obtain the list of concepts that are potentially associated. Notice that entities usually appear in noun phrases, thus, the text chunks to be considered are restricted to these syntactic structures. Finally, in the third step, the concepts whose lexical forms best fit to each text chunk are selected to generate the corresponding semantic annotation.

#### Knowledge resources

As our method relies on the SA of both the user requirement specifications and the web resource metadata, we need to establish the reference KRs from which concepts are brought. Unfortunately, a unique comprehensive ontology for this application domain does not exist, and therefore we need to combine several existing resources. For this purpose, we have selected as main KR the reference ontologies of BioCatalogue (i.e., ^*my*^Grid ontologies) and EDAM Ontology [27], that improves the annotations of the ^*my*^Grid ontologies. We have also used the whole UMLS Meta-thesaurus (version 2010AA) to cover the concepts about procedures, anatomy, diseases, proteomics and genomics. Finally, in order to cover broadly the names of the algorithms and methods involved in Bioinformatics, we have included as concepts the entries of the Wikipedia that have as category some sub-category of the *Bioinformatics* category.

For tagging purposes, all these KRs are loosely integrated into a concept repository which consists of an inventory of concepts, their taxonomical relationships (i.e., *is*_*a* relationship) and the lexical variants associated with each concept (e.g., alternative labels, synonyms, and so on) [28].

#### Normalization through semantic annotation

In order to reconcile the user’s requirements and the resources, we need to normalize their representation under a well-defined semantic space. This normalization process involves the annotation of all the descriptions with the concepts of the unified knowledge resource KR. The purpose of the annotation process is to identify the best-suited concepts for each description found in either web resource metadata or user-requirements specification. To achieve this, we have adopted the automatic annotation method presented in [29], which was tested within the CALBC competition [30]. As mentioned before, this process consists of a mapping function between each text chunk, denoted with *T*, and the lexical variants of each KR concept, denoted with *lex*(*C*). This function is defined as follows:

$$\text{sim}(C,T)=\text{ma}x_{S\in \text{lex}(C)}\left[\frac{\text{info}(S\cap T)-\text{info}(S-T)}{\text{info}(S)}\right]$$

The set lex(C) is a set of lexical strings associated to the concept C. The operation "S intersect T" means the set of tokens both strings S and T share. This function measures the information coverage of *T* with respect to each lexical variant of a concept *C*. Notice that we assume that text chunks and lexical strings are represented as bags of words. Information is measured with an estimation of the string words entropy:

$$\text{info}(S)=-\underset{w\in S}{\sum }\text{log}(P(w|\text{Background}))$$

We have estimated word probabilities over the whole Wikipedia as background.

All these definitions are inspired by the information-theoretic matching function presented in [31] and the word content evidence defined in [32].

The set of annotations associated to each text chunk *T* are those concepts that maximize both *sim*(*C*,*T*) and the word coverage of *T*. That is, the system selects the top ranked concepts whose lexical variants best cover the text chunk *T*. In order to avoid spurious and incomplete annotations, a minimum threshold for *sim*(*C*,*T*) is required (usually above 0.7).

From the annotations set of each description, we define a semantic vector weighted by the *tf*∗*idf* score, where *tf*(*C*) is the frequency of the concept *C* in the description and *idf*(*C*) is calculated as follows:

$$\text{idf}(C)=\text{ma}x_{S\in \text{lex}(C)}\text{info}(S)$$

**Figure 4:**
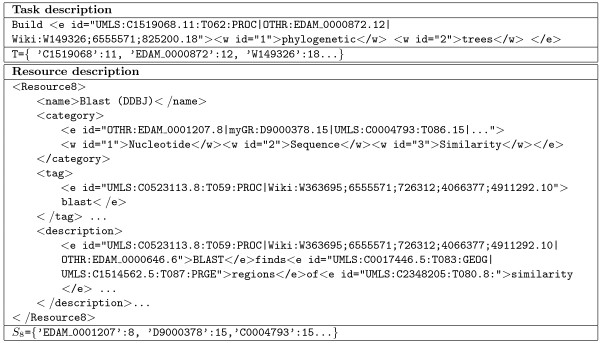
**Semantic annotation of a task and a resource description.** This table shows the semantic annotation and the corresponding semantic vector of the task “build phylogenetic trees” and a fragment of the description of the resource “Blast”. We have used the IeXML notation [33] to show the generated annotations.

Considering the concept reference formats of Table 9, the annotations generated for the example task described as “Build phylogenetic trees”, the metadata of the web resource Blast (DDBJ), and their semantic vectors are shown in Figure 4.

**Table 9:** Concept reference formats

**Source**	**Concept reference format**	**Comment**
UMLS	UMLS:C<number>: STypes	STypesare the semantic types associated to UMLS concepts (e.g. Disease, Protein, etc.)
Wikipedia	Wiki:W<number>: Categs	Categs are the categories associated to the page entry of the referred concept.
myGRID	myGR:D<number>:	These concepts are extracted from the myGRID ontologies.
EDAM	EDAM_<number>:	These concepts are extracted from the EDAM ontology.

This table shows the concept reference formats used for the different semantic sources. The generic format for a reference is Source:ConceptID:SemanticTags.

#### Facets extraction

In this work, we assume that resource descriptions usually lack facet-like metadata, which is very helpful to define user requirements. This kind of information can be implicitly found in the textual resource descriptions, and therefore some kind of information extraction must be performed to obtain those implicit facets. For example, descriptions may contain information about the inputs and the outputs of a resource, the algorithm behind a resource, or the species involved in a public database. We use two techniques to extract information about facets: *(i)* extraction patterns and *(ii)* use of the annotation semantic types. Thus, each facet has associated a set of extraction patterns and a set of semantic types related to it. 

1. **Extraction patterns**. Extraction patterns are applied over the semantically annotated descriptions in order to identify the relevant concepts of each facet. After inspecting some resources, we conclude that the basic extraction pattern for facets is as follows:

(noun-phrase)?¡relation¡E-noun-phrase

Where *E-noun-phrase* denotes any noun phrase containing at least one semantic annotation. Each facet will define the allowed noun phrases and relations of the above generic pattern. For example, Table 3 shows some extraction patterns for the input/output and method facets.

Regarding the example of Figure 4, the patterns of Table 3 extract the following instance:

(Facet¡=¡Output,¡NP¡=¡BLAST,¡R¡=¡finds, ¡C¡=¡{C1514562,¡C2348205})

Where *NP* and *R* are the identified noun phrase and relation of the pattern, and *C* is the set of concepts contained in *E-noun-phrase* part of the pattern.

2. **Semantic types**. The semantic types can also be used to extract information about a facet. Our KR concepts have associated a semantic type which can be very useful to identify relevant information about a facet. For example, a resource can manage information about a specific set of species which are explicitly mentioned in the resource description. Once normalized, their corresponding annotations will have the semantic types of relevant species (e.g., bacteria, virus, mammal, etc.) and, will directly define the species facet. By using their semantic types of annotations the user can define any facet associated to them.

Finally, each facet is represented with the semantic vector obtained from the union of all the concepts associated to that facet in the textual description at hand. This process is applied to user requirements and to the descriptions of the resources.

#### Requirements refinement

Once the requirements have been semantically annotated, the user can tune the annotations to better describe her queries. Each task is represented by a semantic vector which can be modified by the user as follows: 

1. Selection of the most appropriate concepts. The user can choose which concepts of those in the semantic vector are going to be used in the resource retrieval process. In this way, the user disregards wrongly annotated or irrelevant concepts.

2. Selection of more specific concepts. The system prompts to the user a list of narrower concepts to define a more specific query.

3. Selection of more general concepts. The system prompts to the user a list of broader concepts to define a less specific query.

### Web resources retrieval and ranking

The retrieval process of the suitable web resources according to researcher’s requirements is based on the matching between the annotations of the query, including all the facets, and the metadata of the resources. This matching is performed over the semantic vectors associated to them. For example, we could apply the cosine measure to calculate the similarity between web resource descriptions and user requirements, or a concept-based probabilistic model like that presented in [34]. However, these measures do not take into account the relevance of each concept in the context of the tasks to which web resources and requirements are aimed at.

For example, in the queries “define structurally and functionally important domains of the membrane”, “predict gene functions” and “compare functional relationships”, the concept *function* does not have the same relevance. In the first query, *functionally* describes only a characteristic of the domain, in the second one, *function* is the key concept in the query, since it is the object that must be predicted and, finally in the third one, *functional* specifies the type of relationship that must be compared. Therefore, the relevance of the same concept in different queries varies depending on the context.

To be able to exploit this contextual information, our approach is based on a *topic-based ranking model* described in [35]. Using this topic-based model, we can estimate the conditional probabilities of each concept *c* under a pre-defined set of topics *t*_*k*_(1≤*k*≤*n*) (where *n* is the number of topics) which roughly corresponds to bioinformatics generic tasks [36], such as sequence analysis, protein identification, etc.

#### Web resource ranking

Given a query *q*, which consists of a set of concepts *c*_*i*_∈*q* derived from the semantic annotation of a user requirement description, the ranking of resources for a facet *f* is provided with the following probabilities:

$$P(q|ws_{j},f)=\underset{c_{i}\in q}{\prod }\underset{t_{k}}{\sum }P(c_{i}\in f|t_{k})\cdot P(t_{k}|ws_{j},f)$$

Here, *P*(*c*_*i*_∈*f*|*t*_*k*_) is the probability of the concept *c* for the facet *f* given the topic *t*_*k*_, and *P*(*t*_*k*_|*ws*_*j*_,*f*) is estimated with the joint probability of resource *ws*_*j*_ and the task *t*_*k*_ distributions for the facet *f*.

The final similarity between a faceted query *q* and a web resource *ws*_*j*_ is given by the linear combination of the probabilities of the facets in the query.

$$P(q|ws_{j})=\alpha \cdot P(q|ws_{j})+\underset{f}{\sum }\beta_{f}\cdot P(q|ws_{j},f)$$

where

$$\alpha +\underset{f}{\sum }\beta_{f}=1$$

Top ranked resources according to these probabilities are deemed the most appropriate for fulfilling the user requirement query.

### Evaluation

Given a GS, we have evaluated the results obtained for each one of the queries from our query pool with the precision, recall and F-measure. These measures have been calculated as follows:

$$\begin{array}{l} \text{precision}=\frac{\left|\text{relevant}\text{\_}\text{resources}\cap \text{retrieved}\text{\_}\text{resources}\right|}{\left|\text{retrieved}\text{\_}\text{resources}\right|}\\ \text{recall}=\frac{\left|\text{relevant}\text{\_}\text{resources}\cap \text{retrieved}\text{\_}\text{resources}\right|}{\left|\text{relevant}\text{\_}\text{resources}\right|}\\ F=\frac{2\cdot \text{precision}\cdot \text{recall}}{\text{precision}+\text{recall}} \end{array}$$

#### Facets extraction evaluation

The evaluation of the facets extraction method has been carried out for each one of facets by calculating the precision, recall and F-measure as explained next.

For a given facet *F* (e.g., input) we denote with *tags*(*F*) the BioCatalogue tags in the GS assigned to *F*, and with *concepts*(*F*) the automatically extracted concepts for facet *F*. Each tag *t*∈*tags*(*F*) has associated the set of resources annotated with it for the facet *F*, which is denoted with *resources*_*F*_(*t*).

Similarly, each concept *c*∈*concepts*(*F*) has associated the set of resources having *c* as value of the facet *F*, denoted as above.

We calculate precision and recall for each pair (*t*,*c*), *t*∈*tags*(*F*) and *c*∈*concepts*(*F*), as follows:

$$\begin{array}{ll} P_{F}(t,c) & =\frac{\text{resource}s_{F}(t)\cap \text{resource}s_{F}(c)}{\text{resource}s_{F}(c)}\\ R_{F}(t,c) & =\frac{\text{resource}s_{F}(t)\cap \text{resource}s_{F}(c)}{\text{resource}s_{F}(t)}\\ F_{F}(t,c) & =2\cdot \frac{P_{F}(t,c)\cdot R_{F}(t,c)}{P_{F}(c,t)+R_{F}(c,t)} \end{array}$$

The global precision and recall is calculated as a macro-average over the best (*t*,*c*) mappings, which is defined as:

$$\begin{array}{ll} P_{F} & =\underset{t\in \text{tags}(F)}{\sum }P(t,\text{argma}x_{c\in \text{concepts}(F)}(F_{F}(t,c))\cdot \frac{1}{\left|\text{tags}(F)\right|}\\ R_{F} & =\underset{t\in \text{tags}(F)}{\sum }R(t,\text{argma}x_{c\in \text{concepts}(F)}(F_{F}(t,c))\cdot \frac{1}{\left|\text{tags}(F)\right|} \end{array}$$

## Competing interests

The authors declare that they have no competing interests.

## Authors’ contributions

MP developed the framework of the presented discovery method, conducted the experiments and wrote the first draft of the article. RB developed the semantic annotator and provided subject matter expertise critical to the development of the retrieval model. IS developed the interface of BioUSeR. MJ provided useful and very interesting suggestions to improve the design and development of BioUSeR as well as to improve the structure and content of this article. RB and MJ supervised and coordinated the project. All authors read and approved the final manuscript.
